# Phylogenetic analyses and antimicrobial resistance profiles of *Campylobacter* spp. from diarrhoeal patients and chickens in Botswana

**DOI:** 10.1371/journal.pone.0194481

**Published:** 2018-03-21

**Authors:** Stefan P. W. de Vries, Moses Vurayai, Mark Holmes, Srishti Gupta, Michael Bateman, David Goldfarb, Duncan J. Maskell, Maitshwarelo Ignatius Matsheka, Andrew J. Grant

**Affiliations:** 1 Department of Veterinary Medicine, University of Cambridge, Cambridge, United Kingdom; 2 Biological Sciences Department, University of Botswana, Gaborone, Botswana; 3 Department of Paediatrics, Division of Infectious Disease, and Department of Pathology and Molecular Medicine, McMaster University, Hamilton, Ontario, Canada; Cornell University, UNITED STATES

## Abstract

*Campylobacter* spp. are a leading cause of bacterial enteritis worldwide, including countries in Africa, and have been identified by the World Health Organisation (WHO) as one of the high priority antimicrobial resistant pathogens. However, at present there is little knowledge on the prevalence, molecular epidemiology or antimicrobial susceptibility of *Campylobacter* spp. isolates in Botswana, both in patients and in the zoonotic context. Some data indicate that ~14% of diarrhoeal disease cases in a paediatric setting can be ascribed to *Campylobacter* spp., urging the need for the magnitude of *Campylobacter*-associated diarrhoea to be established. In this survey, we have characterised the genomic diversity of *Campylobacter* spp. circulating in Botswana isolated from cases of diarrhoeal disease in humans (n = 20) and from those that colonised commercial broiler (n = 35) and free-range (n = 35) chickens. Phylogeny showed that the *Campylobacter* spp. isolated from the different poultry and human sources were highly related, suggesting that zoonotic transmission has likely occurred. We found that for *Campylobacter* spp. isolated from humans, broilers and free-range chickens, 52% was positive for *tetO*, 47% for *gyrA*-T86I, 72% for *bla*_*OXA-61*_, with 27% carrying all three resistance determinants. No 23S mutations conferring macrolide resistance were detected in this survey. In summary, our study provides insight into *Campylobacter* spp. in poultry reservoirs and in diarrhoeal patients, and the relevance for treatment regimens in Botswana.

## Introduction

Foodborne zoonoses, especially diarrhoeal diseases, are an important cause of morbidity and mortality worldwide, but the epidemiology of diarrhoeal disease in Botswana is poorly understood [1]. Cryptosporidium, *Salmonella*, *Shigella*, Rotavirus and Adenovirus have been associated with past gastroenteritis outbreaks [1]. HIV is endemic in Botswana; according to UNAIDS HIV AIDS estimates in 2016, 350,000 people in Botswana are living with HIV, with 22.2% adult HIV prevalence [2]. Diarrheal disease is a major cause of mortality and morbidity among HIV-infected patients and HIV is a predisposing factor to diarrhoeal disease and associated complications [1].

Infection by *Campylobacter jejuni* is considered to be the most prevalent cause of bacterial diarrhoeal disease worldwide, responsible for ~500 million cases of gastroenteritis per year [3]. The transmission chain of *Campylobacter* spp. is not completely defined but chickens are considered to be the major vehicle for transmission to humans. Additional sources of infection are likely to include red meat, unpasteurised milk and contaminated water. In developing countries, *Campylobacter* spp. infection is common in early childhood owing to poor sanitation and close human contact with animals [4]. With age, infection rates decline, fewer infections are associated with diarrhoea, and the duration and magnitude of convalescent excretion of *Campylobacter* spp. is reduced [5]. Although the severity of *Campylobacter* spp. infection in adults is different between developed and developing countries, the clinical signs of infection in adults in developing countries appear to be similar to those in developed countries [6]. Acute signs range from protracted watery diarrhoea to bloody diarrhoea with fever, abdominal cramps, and the presence of faecal leukocytes [4, 7]. Although the vast majority of cases are self-limiting, there is emerging data in developing countries that even very common ‘asymptomatic’ infections (those without diarrhoea) is associated with reduced linear growth (low height for age) [8].

Limited studies have been conducted in developing countries that report on *Campylobacter* spp. in both humans and animals, in particular genotypic information [9]. One study carried out at the two referral hospitals in Botswana found that 14% of children admitted with acute gastroenteritis had *Campylobacter* spp. detected in their stool [10].

Most *Campylobacter* spp. infections are self-limiting and treatment is usually supportive. However, effective antimicrobial therapy is critical for people with severe or prolonged campylobacteriosis, for the elderly, for the young or for the immunocompromised [9, 11, 12]. The development of resistance by the bacterium obviously limits treatment options in humans and other animals [11]. A recent study of *Campylobacter* spp. in the private health care sector in South Africa found a high prevalence of resistance to the fluoroquinolones, macrolides and tetracycline, which are drugs in the first-line treatment of *C*. *jejuni* and *C*. *coli* [9]. In recent years, isolates from both developed and developing countries have shown resistance to several antimicrobials, including fluoroquinolones, tetracyclines, beta-lactams, aminoglycosides and macrolides [7, 9, 11, 13], which are the most frequently used antimicrobials for the treatment of campylobacteriosis [6, 7, 9, 11–14]. This has led the World Health Organisation in 2017 to list *Campylobacter* spp. as one of the six high priority antimicrobial resistant pathogens [15]. Antimicrobial usage in both animal agriculture and human medicine can influence the development of antibiotic-resistance in *Campylobacter* spp. [13]. It is thought that the unrestrained use of antimicrobials in developing countries, in particular, has contributed to increased antimicrobial resistance in *Campylobacter* spp. [6, 7, 11]. In some countries, antimicrobials are still used as growth promoters as opposed to therapeutic agents [11]. As a zoonotic pathogen, antimicrobial-resistant isolates of *Campylobacter* spp. can be transferred to humans *via* contaminated food, water or milk. The use of fluoroquinolones in food animals, in particular chickens, has been associated with increases in resistant *Campylobacter* spp. causing disease in humans [16]. However, little is understood regarding the potential contribution of this source of antimicrobial resistance in the African context.

Genotypic information about *Campylobacter* spp. in developing countries is lacking [17]. Currently there are no available data on the prevalence, molecular epidemiology or antimicrobial susceptibility of *Campylobacter* spp. isolates in Botswana, neither in patients nor in poultry reservoirs. As such, this is the first survey conducted in Botswana with the aim to characterise *Campylobacter* spp. isolated from diarrhoeal patients, and from free-range and broiler chickens to determine the genetic diversity and the presence of antimicrobial resistance genes.

## Results and discussion

### Diversity of *Campylobacter* spp.

*Campylobacter* spp. were isolated from diarrhoeal paediatric patients admitted to the Princess Marina Hospital in Gaborone, Botswana, and from caecal samples collected from commercial broiler and free-range chickens. The chickens were purchased from various poultry farms within the Gaborone catchment area, which is located close to the border with South Africa. Chickens were obtained from five sites with commercial broilers and eight sites with free-range chickens.

Genomes were sequenced using Illumina HiSeq or MiSeq technologies for 20 *Campylobacter* spp. isolates from human diarrhoeal patients and 70 isolates from chickens, of which 35 were from commercial broilers and 35 were from free-range chickens. To assess which *Campylobacter* species each isolate belonged to, an initial comparative genome analysis was performed in by pairwise average nucleotide identity (ANI) BLAST analysis in JSpeciesWS [18] using the generally accepted ANI species cut-off of 94% [19] (S1 Table). Human and free-range chicken isolates were predominantly *C*. *jejuni*, with 19 human isolates being *C*. *jejuni* (95%) and one being *C*. *coli* (5%), while 29 (82.8%) free-range chicken isolates were *C*. *jejuni* and 6 (17.1%) were *C*. *coli*. Out of the 35 commercial broiler isolates, 14 (40.0%) were *C*. *jejuni* and 21 (60.0%) were *C*. *coli* (S1 Table).

Mutilocus sequence typing (MLST) identified 19 sequence-types (STs) for *C*. *jejuni* and 7 for *C*. *coli* (S1 Table). Two novel STs were identified for *C*. *jejuni*; ST9024 and ST9027, which both belong to clonal complex ST354. Three novel STs were found for *C*. *coli*; ST9025 (clonal complex ST1150), and ST9026 and ST9028 that both belong to clonal complex ST828.

Noteworthy, 24 out of 28 *C*. *coli* isolates belonged to clonal complex ST2828 (S1 Table), indicating that, based on MLST, the *C*. *coli* isolates are genetically more conserved. In recent Catalonian (north-east Spain) and Italian studies, the predominant clonal complex of *C*. *coli* isolates was also ST828 [20, 21]

### Phylogenetic analyses of *Campylobacter* spp.

**Fig 1:**
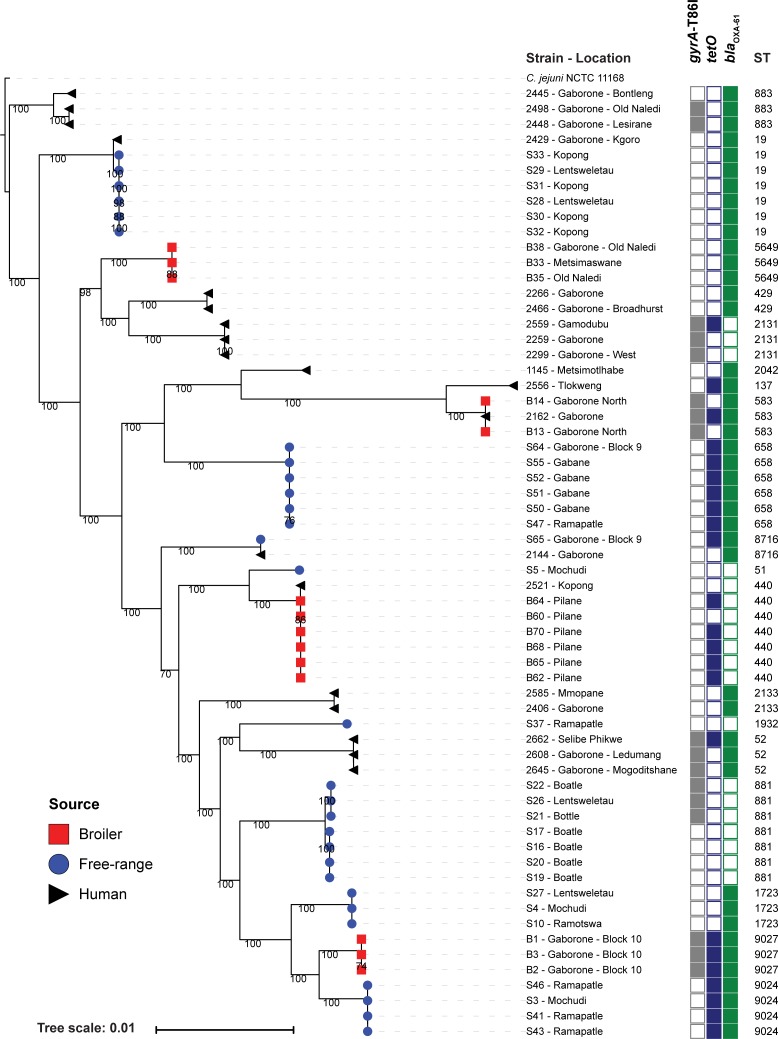
Phylogeny and AMR profile of *C*. *jejuni* isolates from this study. Core genome maximum likelihood phylogeny of *C*. *jejuni* isolates visualised in the interactive Tree of life tool (iTol) [23]. The tree was rooted on isolate 2445 to facilitate comparison with the SNP-based phylogeny shown in S1 Fig. Clustering of isolates was found to be in accordance between core genome and SNP-based phylogenies (S1 Fig). Clustering of isolates belonging to the same ST was consistent. Shown for each isolate are: isolate identifier, the geographic location of isolation, presence of AMR determinants and ST.

**Fig 2:**
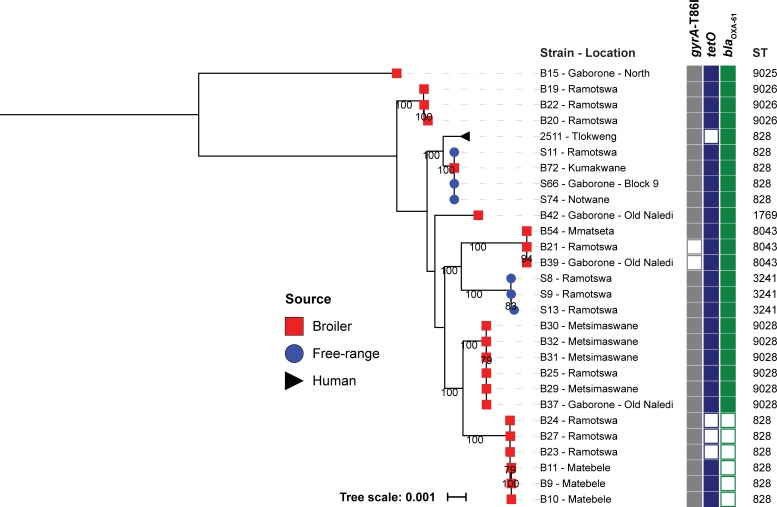
Phylogeny and AMR profile of *C*. *coli* isolates from this study. Core genome maximum likelihood phylogeny of *C*. *coli* isolates visualised in the iTol [23]; the tree was rooted with isolate B15 as an outgroup. Clustering of isolates was found to be in accordance between core genome and SNP-based phylogenies (S2 Fig). Isolates belonging to the same ST clustered together. Shown for each isolate are: isolate identifier, the geographic location os isolation, presence of AMR determinants and ST.

Phylogeny was built separately for *C*. *jejuni* (Fig 1) and *C*. *coli* (Fig 2) based on core genome alignments, which include 1107 or 1197 genes respectively. In addition, phylogeny was also assessed using extracted single nucleotide polymorphisms (SNPs) derived from a reference-based alignment; *C*. *jejuni* NCTC11168 [22] was used as a reference (S1 Fig and S2 Fig). Clustering of isolates in both core genome- and SNP-based phylogenies was identical, indicating that both methods successfully extracted the existing phylogenetic signal. It is noteworthy that MLST seems to be an effective indication of related lineages as isolates belonging to the same ST clustered together. Each clade, as defined by ST, appears to contain very little genetic diversity within the clade compared to the differences between clades. These results suggest that the employed methods provide a clear indication of the relatedness of the isolates. However, due to the genetic promiscuity of *Campylobacter* spp., the trees are unlikely to represent the true phylogenies.

Our data show that isolates from across the phylogenies of each Campylobacter spp. are present in broilers, free-range chickens and humans. As anticipated, strains from the same sample set/location were closely related. However, there is also evidence of (closely) related strains in different locations, suggesting that geographic spread may have occurred. Regarding the possibility of geographic spread of *C*. *jejuni*, we observed that isolate S26 from a free-range chicken in Lentsweletau was present in a cluster with isolates from free-range chickens from Boatle that were isolated six months previously. S3 from a free-range chicken in Mochudi was present in a cluster with isolates from free-range chickens from Ramapatle isolated fourteen months later. S28 and S29 from free-range chickens in Lentsweletau are present in a cluster with isolates from free-range chickens from Kopong isolated one month later. S50, S51 and S55 from free-range chickens in Gabane are present in a cluster with isolates from free-range chickens from Gaborone—block 9 (S64) isolated ~2 weeks later and Ramapatle (S47) isolated ~1 week previous. S4, S10 and S27 from free-range chickens, each from a different geographical location and from a different sampling time (spanning seven months) form a cluster (S1 Table, Fig 1 and S1 Fig).

Regarding the possibility of geographic spread of *C*. *coli*, we observed that isolate B39 from a broiler chicken in Gaborone—Old Naledi was present in a cluster with isolates from broiler chickens in Ramotswa (B21) isolated fifteen months previously and Mmatseta (B54) isolated three months later. Isolates B23, B24 and B27 from broiler chickens in Ramotswa were present in a cluster with isolates from broiler chickens in Matebele (B9, B10 and B11) isolated four months later. Isolates B30, B31 and B32 from broiler chickens in Metsimaswane were present in a cluster with isolates from broiler chickens in Ramotswa (B25) and Metsimaswane (B29) isolated on the same day and Gaborone—Old Naledi (B37) isolated fifteen months later. *C*. *coli* B15 isolated from a broiler in Gaborone–North, ST9025, a novel ST belonging to clonal complex ST1140, is distant to the other *C*. *coli* isolates in this study (Fig 2 and S2 Fig). Pair-wise ANI analyses with the other *C*. *coli* isolates in our study confirmed that B15 belongs to the *C*. *coli* species, *i*.*e*. all were >94% ANI, which is generally considered the species cut-off value [19].

We observed limited mixing of strains between broiler and free-range chickens, as evidenced by most branches of the phylogeny only having strains from a single host, with the exception of *C*. *coli* isolates S11 from a free-range chicken in Ramotswa, S66 from a free-range chicken in Gaborone Block 9 isolated nineteen months later and S74 from a free-range chicken in Notwane isolated eighteen months later were present in a cluster with *C*. *coli* isolates from a broiler chicken B72 isolated nineteen months later from Kumakwane and a human isolate 2511 from Tlokweng (date of isolation not recorded, but before the isolates from the chickens were collected) (S1 Table, Fig 1 and Fig 2).

For *C*. *jejuni* we observed human isolates that were highly related to chicken isolates. This included *i*) human isolate 2144 from Gaborone and S65 isolated from a free-range chicken in Gaborone Block 9, *ii*) human isolate 2162 (Gaborone) is related to broiler isolates B13 and B14 from Gaborone North, *iii*) human isolate 2521 is related to broiler isolates B60, B62, B64, B65, B68 and B70 (Fig 1 and S1 Fig).

### Comparative analyses of *Campylobacter* spp. from Botswana with other African isolates

**Fig 3:**
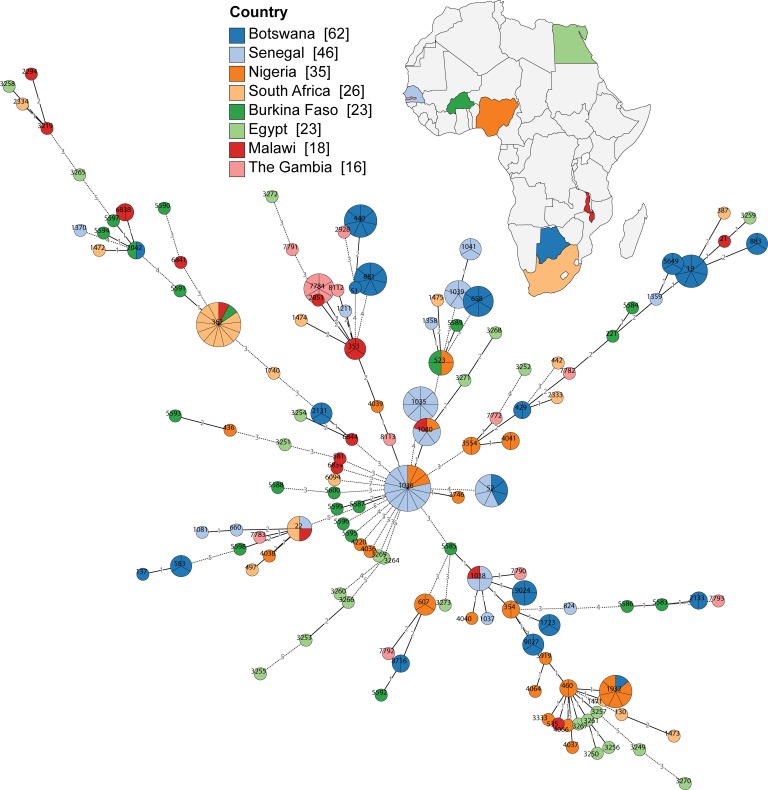
MLST of *C*. *jejuni* isolates from this study and other African *C*. *jejuni* isolates. Minimum spanning tree generated from *C*. *jejuni* allelic profiles visualised in GrapeTree [25], which included African *C*. *jejuni* isolates that were available in the PubMLST database [24] (accessed in January 2017). Isolates are colour-coded according to country and labelled with STs. The number of isolates per node (ST) are indicated through pie-charts. The number of nucleotide differences between STs is depicted at the branches. If STs differ by more than 2 nucleotides, branches are truncated (dashed lines). More details can be found in S1 Table and in the text.

**Fig 4:**
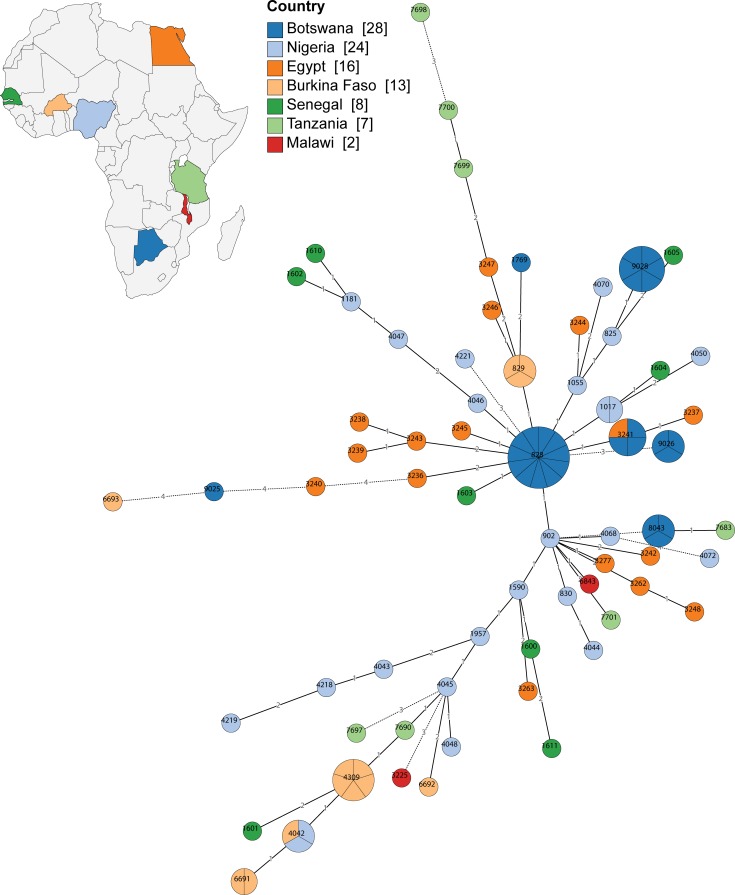
MLST of *C*. *coli* isolates from this study and other African *C*. *coli* isolates. Minimum spanning tree generated from *C*. *coli* allelic profiles visualised in GrapeTree [25], which included African *C*. *coli* isolates that were available in the PubMLST database [24] (accessed in January 2017). Isolates are colour-coded according to country and labelled with STs. The number of isolates per node (ST) are indicated through pie-charts. The number of nucleotide differences between STs is depicted at the branches. If STs differ by more than 2 nucleotides, branches are truncated (dashed lines). More details can be found in S1 Table and in the text.

The distribution of *Campylobacter* spp. isolates from Botswana was analysed with other African isolates using multi locus sequence type (MLST) allelic profiles available from the PubMLST database [24] (accessed in January 2017). Minimum spanning trees generated and visualised in GrapeTree [25] were used to assess the phylogenetic relatedness amongst African isolates; trees were built separately for *C*. *jejuni* (Fig 3) and *C*. *coli* (Fig 4) isolates. STs represented by Botswanan isolates predominantly comprised isolates from Botswana only, with the exception of *i*) *C*. *jejuni* ST1932 that comprised 6 Nigerian and 1 Botswanan isolate, *ii*) *C*. *jejuni* ST52 comprised 4 isolates from Senegal and 3 isolates from Botswana, *iii*) *C*. *coli* ST3241 with 3 isolates from Botswana and 1 from Egypt. In line with the dispersed distribution of African *C*. *jejuni* and *C*. *coli* isolates across the MLST-based tree, Botswanan *Campylobacter* spp. isolates were also widely distributed across the tree (Fig 3 and Fig 4). Several *C*. *jejuni* STs are located at the outside of the tree, *e*.*g*. ST137 (human isolate 2556) and ST583 (human isolate 2162, and broiler isolates B13 and B14) that belong to clonal complex ST45, and the clonal complex ST21 isolates that belong to ST19, ST883 and ST5649 that comprises human, free-range and broiler isolates (S1 Table and Fig 3). Some of the commercial broiler *C*. *coli* isolates appear to be more distantly related to other African *C*. *coli* isolates, *i*.*e*. B15 (ST9025) and isolates belonging to ST9028 (B25, B29, B30, B31 and B27) (S1 Table and Fig 4). The data highlight the diversity of *Campylobacter* spp. in Botswana and indicate both local and global strains, confirming previous MLST studies with *C*. *jejuni* and *C*. *coli* isolates from poultry, cattle and humans in Nigeria [17].

### Antimicrobial resistance profiles derived from whole genome sequence data

Fluoroquinolones, tetracyclines, beta-lactams, aminoglycosides and macrolides are the most frequently used antimicrobials for the treatment of campylobacteriosis [6, 7, 9, 11–14]. In *Campylobacter* spp., as in other Gram-negative bacteria, the main mechanism of fluoroquinolone resistance is chromosomally mediated through mutation of *gyrA* by alteration of codon 86 from threonine to isoleucine [6]. *Campylobacter* spp. carrying the Thr-86-Ile change in the GyrA subunit of DNA gyrase can persist in the absence of antimicrobial selection pressure [11]. In *Campylobacter* spp. resistance to tetracycline is primarily mediated by a ribosomal protection protein (*tetO*) that is transferred as a plasmid-encoded gene or on the chromosome where it is not self-mobile [6, 9, 14]. Resistance to ampicillin and other beta-lactams is widely reported among *Campylobacter* spp. isolated from humans and poultry [26, 27]. A significant knowledge gap still exists concerning the molecular basis of beta-lactam resistance in *Campylobacter* spp. [28]. Resistance to beta-lactams in *Campylobacte*r spp. can be mediated by OXA-61 beta-lactamase, which is encoded by the *bla*_OXA-61_ gene [29], but other beta-lactamases also exist [26]. Aminoglycoside (gentamicin) resistance occurs through the presence of genes including *aph(2”)-IF*, *aac/aphD*, *aph(2”)-Ig* and *aacA4* [30]. High-level macrolide resistance is mediated through mutation of the 23S rRNA [6].

We performed an *in silico* assessment for mutations conferring antimicrobial resistance and for the presence of antimicrobial resistance genes. For both *C*. *jejuni* and *C*. *coli* isolates there was a noticeable presence of *tetO* (52%), *gyrA*-T86I (47%) and *bla*_*OXA-61*_ (72%) (Fig 1, Fig 2, Table 1 and S1 Table). We found no evidence of other antimicrobial resistance genes (as described in Materials and Methods). In comparison, a recent WGS-based study of 16 Catalonian (north-east Spain) broiler *Campylobacter* spp. isolates (12 *C*. *jejuni* and 4 *C*. *coli*) revealed higher prevalence of *tetO* (81%) and *gyrA*-T86I (100%) [20].

**Table 1:** Frequency of antimicrobial resistance genes (*tetO*, *gyrA-*T86I and *bla*_*OXA-61*_) in *C*. *jejuni* and *C*. *coli* from broiler, free-range chickens and human clinical isolates.

***C*. *jejuni***	**Broiler**	**Free range**	**Human**	**Total**
*blaOXA-61*	8/14 (57.1%)	20/29 (68.9%)	15/19 (78.9%)	43/62 (69.4%)
*tetO*	8/14 (57.1%)	11/29 (37.9%)	4/19 (21%)	23/62 (37.1%)
*gyrA*-T86I	5/14 (35.7%)	3/29 (10.3%)	9/19 (47.4%)	17/62 (27.4%)
*blaOXA-61* + *tetO*	3/14 (21.4%)	11/20 (37.9%)	3/19 (15.8%)	17/62 (27.4%)
*blaOXA-61* + *gyrA*-T86I	5/14 (35.7%)	0/29 (0%)	6/19 (31.6%)	11/62 (17.7%)
*tetO* + *gyrA*-T86I	3/14 (21.4%)	0/29 (0%)	3/19 (15.8%)	6/62 (9.7%)
*blaOXA-61* + *tetO* + *gyrA*-T86I	3/14 (21.4%)	0/29 (0%)	2/19 (10.5%)	5/62 (8.1%)
None	1/14 (7.1%)	6/29 (20.7%)	1/19 (5.3%)	8/62 (12.9%)
				
***C*. *coli***	**Broiler**	**Free range**	**Human**	**Total**
*blaOXA-61*	15/21 (71.4%)	6/6 (100%)	1/1 (100%)	22/28 (78.6)
*tetO*	18/21 (85.7%)	6/6 (100%)	0/1 (0%)	24/28 (85.7%)
*gyrA*-T86I	19/21 (90.5%)	6/6 (100%)	1/1 (100%)	26/28 (92.9%)
*blaOXA-61* + *tetO*	15/21 (71.4%)	6/6 (100%)	0/1 (0%)	21/28 (77.8%)
*blaOXA-61* + *gyrA*-T86I	13/21 (61.9%)	6/6 (100%)	1/1 (100%)	20/28 (71.4%)
*tetO* + *gyrA*-T86I	16/21 (76.2%)	6/6 (100%)	0/1 (0%)	22/28 (78.6%)
*blaOXA-61* + *tetO* + *gyrA*-T86I	13/21 (61.9%)	6/6 (100%)	0/1 (0%)	19/28 (67.9%)
None	0/21 (0%)	0/6 (0%)	0/1 (0%)	0/28 (0%)

In this study, mutation of *gyrA* was limited to T86I, confirming that this is the most common *gyrA* mutation. This is also in line with studies conducted in Portugal, Catalonia and Poland in which the only detected *gyrA* mutation was T86I [20, 31, 32].

Mutations of the 23S rRNA gene that confer macrolide resistance were not detected in the Botswanan *Campylobacter* spp. isolates reported in this study, indicating that prevalence of macrolide resistance is low in Botswana. This is in line with a Polish study, which detected the A2075G 23S rRNA mutation only in 8 out of 802 poultry isolates [32]. However, a Catalonian study found the A2075G 23S rRNA mutation in 3 out of 4 tested *C*. *coli* isolates but not in the *C*. *jejuni* isolates [20], whereas an Italian study observed the A2075G 23S rRNA mutation in 49 out of 197 tested *Campylobacter* spp. isolates [21].

We found that *tetO* was more prevalent in *C*. *coli* (24/28; 85.7%) than *C*. *jejuni* (23/62; 37.1%) (P = 0.0003). Also, *gyrA*-T86I was more prevalent in *C*. *coli* (26/28; 92.9%) than *C*. *jejuni* (17/62; 27.4%) (P = 6.93e-08) (Table 1). Our analyses showed that *tetO* was more prevalent in *Campylobacter* spp. isolated from broilers than from humans (P = 0.0021) (Table 1). We observed that the *gyrA*-T86I mutation was more prevalent in *Campylobacter* spp. isolated from broilers than from free-range chickens (P = 0.0082) (Table 1). Also, we found that *bla*_*OXA-61*_ was widely distributed in *C*. *jejuni* 43/62 (69.4%) and *C*. *coli* 22/28 (78.6%) (Table 1). This is in line with a survey conducted in the United Kingdom, in which 52% of poultry associated *Campylobacter* spp. isolates were ampicillin-resistant, of which 92% carried *bla*_*OXA-61*_ [26].

*C*. *coli* isolates had higher numbers of individual antimicrobial resistance genes, hence they also had a higher proportion of combinations of resistance genes. All three resistance genes i.e. *tetO*, *gyrA*-T86I and *bla*_*OXA-61*_ were present in 5/62 (8.1%) of *C*. *jejuni* isolates and 19/28 (67.9%) of *C*. *coli* isolates (Table 1). This finding supports previous studies that have suggested that *C*. *coli* has increased resistance due to exposure to a larger number of antimicrobial agents compared to *C*. *jejuni* [12, 14]. None of the *C*. *coli* isolates were negative for any of the antimicrobial resistance genes 0/28 (0%), while 8/62 (12.9%) of the *C*. *jejuni* isolates were negative (Table 1). Interestingly, all six *C*. *coli* isolates from the free-range birds contained all three resistance genes.

## Conclusion

Here we report on the genomic diversity of circulating *Campylobacter* spp. causing diarrhoeal disease in humans and of colonised chickens in Botswana. Phylogeny showed that the *Campylobacter* spp. isolated from the different poultry and human sources were highly related, suggesting that zoonotic transmission is likely.

In a large proportion of *Campylobacter* spp. isolated from humans, broilers and free-range chickens in this study we detected *tetO* (52%), *gyrA*-T86I (47%) and *bla*_*OXA-61*_ (72%) genes. Our study has identified isolates carrying multiple drug resistance genes, *i*.*e*. 27% of the isolates carried all three above mentioned resistance determinants. The *C*. *coli* isolates analysed in our study had a higher prevalence of the individual antimicrobial resistance genes when compared to *C*. *jejuni* isolates, which is in line with other studies [20, 21, 31, 32]. The whole genome sequence data indicates that *Campylobacter* spp. isolated from humans and chickens in Botswana have the potential to be resistant or multiply resistant to a number of antimicrobial classes, however we did not test specifically for phenotypic antimicrobial resistance. The presence of antimicrobial resistance genes to first line treatment drugs is a concern. Collectively, the data suggest that the efficacy of using current treatments for treating human campylobacteriosis and for the treatment or use as growth promoters in chickens should be evaluated.

Our analyses did not identify any *Campylobacter* spp. carrying 23S rRNA gene mutations that confer macrolide resistance, suggesting that erythromycin or azithromycin may still be an effective treatment. In addition, aminoglycosides such as gentamycin may be an alternative option as we found no evidence of *aphA-3* and *aacA4* genes.

Importantly, this survey provides detailed insight into the phylogeny of and antimicrobial resistance profiles of *Campylobacter* spp. in Botswana. Further studies that cover a larger geographic study area are necessary to detect emerging resistance patterns and to assess the impact of strategies designed to mitigate antimicrobial resistance.

## Materials and methods

### Ethics statement

This study was reviewed and approved by the institutional review boards of the Botswana Ministry of Health, Gaborone, Botswana–PPME-13/18/1 Vol VII (434); Princess Marina Hospital, Gaborone, Botswana—PPME-13/18/1 Vol VII (434); Health Research and Development Committee, University of Botswana—REF:UBR/RES/IRB/GRAD/394; University of Cambridge, Cambridge, United Kingdom—REF:HBREC.2016.19. Written informed consent was obtained from guardians/parents for all human subjects who had stool samples collected for this study.

### Isolation of *Campylobacter* spp. from commercial and free-range broilers

The chickens were purchased from various poultry farms within the Gaborone catchment area, which is located close to the border with South Africa. Chickens were obtained from five sites with commercial broilers and eight sites with free-range chickens. The dataset included fully grown ready-for-slaughter chickens from poultry farms and free-range chickens obtained from small enterprises; only one farm was sampled in each sample represented in S1 Table. ‘Free-range’ chickens refer to the indigenous breed of chickens kept in Botswana. This type of chicken is slow growing, reaching maturation and ready-for-slaughter at 5–6 months (opposed to the broilers that are slaughtered at 4–5 weeks). These chickens are not housed but are allowed to roam and forage in the farm area during the day, they are only sheltered to protect them from predators at night.

A loop full of caecal sample was streaked on *Campylobacter* Blood-free selective agar; Charcoal-Cefoperazone-Deoxycholate Agar (CCDA) [33], mixed with a selective supplement SR0155 containing cefoperazone 32 mg/l and amphotericin B 10 mg/l of medium (Oxoid).

Isolates were incubated at 37°C for 48 h under microaerobic conditions (Oxygen 5%, Carbon dioxide 5–8%) achieved using GasPak^TM^ EZ *Campylobacter* sachets and incubation chambers (Becton Dickinson and Company, USA). A plate inoculated with *Campylobacter* spp. strain, *C*. *jejuni* ATCC^®^ 33560^TM^ (Microbiologics St. Cloud, USA), was included in every incubation chamber as a control. Typical watery, spreading and convex colonies were picked and screened for *Campylobacter* spp. using Gram stain, catalase and oxidase tests. Gram-negative, curved or seagull wing-shaped, catalase and oxidase positive, bacterial isolates were purified and suspended in cryovials containing plastic beads and 40% glycerol, followed by freezing and storage at -80°C.

### Isolation of *Campylobacter* spp. from human infection cases

Specimens were obtained from diarrhoeal patients from the largest referral hospital in Botswana (Princess Marina Hospital, Gaborone, Botswana) and surrounding clinics. Inclusion criteria include all stools from patients with acute gastroenteritis under the age of 13 years and loose, watery and bloodstained stools from patients over the age of 13 years. Acute gastroenteritis was defined as having >2 loose bowel movements in a 24 h period or vomiting with any loose stools with a maximum duration of 13 days. Exclusion criteria included, discharge from hospital within 7 days of admission and diarrhoea of 14 days or longer. Samples were only collected from patients that had given approval.

Stools collected in Carry Blair transport media (Becton Dickinson and Company, USA) were cultured on the same day on *Campylobacter* selective agar; Columbia agar base with Skirrow’s selective supplement with added vancomycin 10 mg/l, polymixyn B 5 mg/l and trimethoprim 2500 IU/l and 10% sheep blood. Samples were incubated under microaerobic conditions (CampyGen, Oxoid). The weight and volume of the inoculum was not measured for each sample; a loop-full piece of stool, picked from the mucous part of the specimen, was streaked on the selective agar media. Gram-negative curved or seagull wing-shaped, catalase and oxidase positive, bacterial isolates were purified by subculture on new blood agar plates. The isolates were suspended in cryovials containing plastic beads and 40% glycerol, followed by freezing and storage at -80°C.

### Isolation of *Campylobacter* spp. genomic DNA for sequencing

Pure cultures of isolates were individually suspended in saline to match 0.5 MacFarland standard. DNA was extracted from the suspensions using MagNA Pure Compact Nucleic Acid Isolation Kit I (Roche Diagnostics GmbH, Germany) on MagNA Pure Compact automated extraction and purification platform, according to the manufacturer’s instructions (Roche Diagnostics GmbH, Germany).

### Whole genome sequencing

Genome sequencing libraries were prepared using the NEBNext Ultra II DNA library prep kit (New England Biolabs) as described in de Vries *et al*. [34]. The libraries were sequenced using 150 bp paired-end sequencing on the Illumina HiSeq 4000 platform (Genomics core facility at Cancer Research UK) or on the Illumina MiSeq platform using 250–300 bp paired-end sequencing (in-house facility).

### Genome assembly and annotation

The Illumina HiSeq 4000 read files were demultiplexed using the demuxFQ tool (Cancer Research UK) and adapter-trimmed in Cutadapt. *De novo* draft genome assemblies were created using Illumina HiSeq or MiSeq paired-end reads using Spades v3.6.2 [35] and annotated with Prokka [36].

### *In silico*-based MLST phylogeny

MLST was performed using the short-read sequence-typing tool SRST2 [37] with allele sequences derived from the PubMLST database [24] (accessed in January 2017) as reference. Novel allele sequences and STs were submitted to PubMLST database [24]. For comparative analyses with African *Campylobacter* spp. isolates, MLST minimum spanning trees were generated and visualised in GrapeTree [25], which included African *C*. *jejuni* and *C*. *coli* isolates available in PubMLST database [24] (accessed in January 2017). Further examination of the assembled genomes of the *C*. *coli* revealed some anomalies with the MLST genes. In 17 out of 28 *C*. *coli* isolates we observed the presence of an additional MLST gene allele. In all cases, this involved a single gene out of the 7 MLST genes, e.g. in *C*. *coli* 2511 both *gltA*_16 and *gltA*_30 were present and in *C*. *coli* B10 both *gltA*_10 and *gltA*_30 were found. The second allele was consistently detected at low coverage and in small contigs. Collectively, this involved 6 out of the 7 MLST genes: *gltA*, *uncA*, *aspA*, *glyA*, *tkt* and *glnA*. For the *C*. *coli* MLST analyses presented, the high sequence coverage hits were selected. Importantly, such observations were not made in any of the *C*. *jejuni* isolates which were sequenced at the same time, intermingled with the *C*. *coli* isolates, suggesting that this is not related to a sequencing error or contamination. This may be the result of horizontal gene transfer with the species *C*. *coli*.

### Core genome and SNP-based phylogenetic analyses

Core genome phylogenetic analysis was performed in Roary [38] using the Prokka annotation [36]; a 95% nucleotide identity cut-off was applied for inclusion in the core genome. Sequences were aligned using MAFTT [39] and Maximum Likelihood (ML)-phylogeny was reconstructed in RaxML v8.2.9, with GTRCAT approximation and automatic bootstrapping (autoMRE) using rapid bootstrapping mode [40], and visualised in the interactive Tree of life tool (iTol) [23]. For SNP-based phylogenies, sequence reads were aligned against *C*. *jejuni* NCTC11168 [22] as a reference genome using SMALT [41], following default settings to identify SNPs. Extracted SNPs were used to construct ML-phylogenies using RaxML v 7.3.6 (rapid bootstrapping and subsequent ML search using 100 bootstrap replicates) [40].

### *In silico* identification of antimicrobial resistance determinants

The *gyrA* and 23S rRNA gene sequences were extracted from the Prokka genome annotations. The *gyrA* sequence was translated to the protein sequence and aligned. The 23S rRNA gene sequences were directly aligned. Alignments were conducted in in CLC main workbench v7.3.3. For GyrA we assessed aa position 70 for Ala to Thr (not found); amino acid (aa) position 86 Thr to Ile (found and discussed), aa position 90 Asp to Asn (not found). For the 23S rRNA gene we assessed positions 2074 and 2075 of which mutations confer high-level macrolide resistance [42]; no resistance-associated mutations were found. The intergenic region between *cmeR* and *cmeABC* or mutations in the L4 and L22 ribosomal proteins were not assessed as part of this study.

Antimicrobial resistance (AMR) gene detection was conducted through alignment of Illumina sequence reads to a custom AMR gene database, which was built from gene sequences available from the ARG-ANNOT [43] and RESfinder [44] databases. Of note, the compiled AMR gene database also contained *ermB* that is implicated in macrolide resistance [43].

### Statistical analyses

Statistical analyses were performed in R *via* the rpy2 module in Python. Bonferroni-corrected P-values are reported.

### Nucleotide sequence accession numbers

Illumina whole genome sequencing data has been deposited in the European Nucleotide Archive (http://www.ebi.ac.uk/ena) and are available *via* study accession number PRJEB18561.

## Supporting information

S1 Table Overview of *Campylobacter* spp. isolates including; ID, source, sample location, sample date, species and antimicrobial resistance gene (*tetO*, *gyrA-*T86I and *bla*_*OXA-61*_) presence (P) or absence (A), MLST sequence type (ST), allelic profiles and ENA accession numbers. (DOCX)

S1 Fig SNP-based phylogeny and AMR profile of *C*. *jejuni* isolates. SNP-based maximum likelihood phylogeny of *C*. *jejuni* isolates visualised in interactive Tree of life tool (iTol) [S1]. The tree was rooted on reference isolate *C*. *jejuni* NCTC11168 [S2]. Clustering of isolates was found to be in accordance between core genome and SNP-based phylogenies (Fig 1). Clustering of isolates belonging to the same ST was consistent. Shown for each isolate are: isolate identifier, the geographic location of isolation, presence of AMR determinants and ST.
(DOCX)

S2 Fig SNP-based phylogeny and AMR profile of C. coli isolates. SNP-based maximum likelihood phylogeny of *C*. *coli* isolates visualised in interactive Tree of life tool (iTol) [S1]. The tree was rooted on reference isolate *C*. *jejuni* NCTC11168 [S2]. Clustering of isolates was found to be in accordance between core genome and SNP-based phylogenies (Fig 2). Clustering of isolates belonging to the same ST was consistent. Shown for each isolate are: isolate identifier, the geographic location of isolation, presence of AMR determinants and ST.
(DOCX)

## Abbreviations

ANI: average nucleotide identity
CCDA: Charcoal-Cefoperazone-Deoxycholate Agar
iTol: interactive Tree of life
MLST: multilocus sequence type.
