# Supplementary material for: Phylogenetic analyses and antimicrobial resistance profiles of Campylobacter spp. from diarrhoeal patients and chickens in Botswana
Source: PLoS One. 2018 Mar 21;13(3):e0194481. doi: 10.1371/journal.pone.0194481 (PMC5862492; doi:10.1371/journal.pone.0194481)
Supplement: S1 Table — (DOCX) [file pone.0194481.s001.docx]

**Table S1.** Overview of Campylobacter spp. isolates including; ID, source, sample location, sample date, species and antimicrobial resistance gene (tetO, gyrA-T86I and blaOXA-61) presence (P) or absence (A), MLST sequence type (ST) and allelic profiles, and ENA accession numbers.
