# Supplementary material for: Phylogenetic analyses and antimicrobial resistance profiles of Campylobacter spp. from diarrhoeal patients and chickens in Botswana
Source: PLoS One. 2018 Mar 21;13(3):e0194481. doi: 10.1371/journal.pone.0194481 (PMC5862492; doi:10.1371/journal.pone.0194481)
Supplement: S2 Fig — SNP-based maximum likelihood phylogeny of C. coli isolates visualised in interactive Tree of life tool (iTol) [S1]. The tree was rooted on reference isolate C. jejuni NCTC11168 [S2]. Clustering of isolates was found to be in accordance between core genome and SNP-based phylogenies (Fig 2). Clustering of isolates belonging to the same ST was consistent. Shown for each isolate are: isolate identifier, the geographic location of isolation, presence of AMR determinants and ST. (DOCX) [file pone.0194481.s003.docx]

**
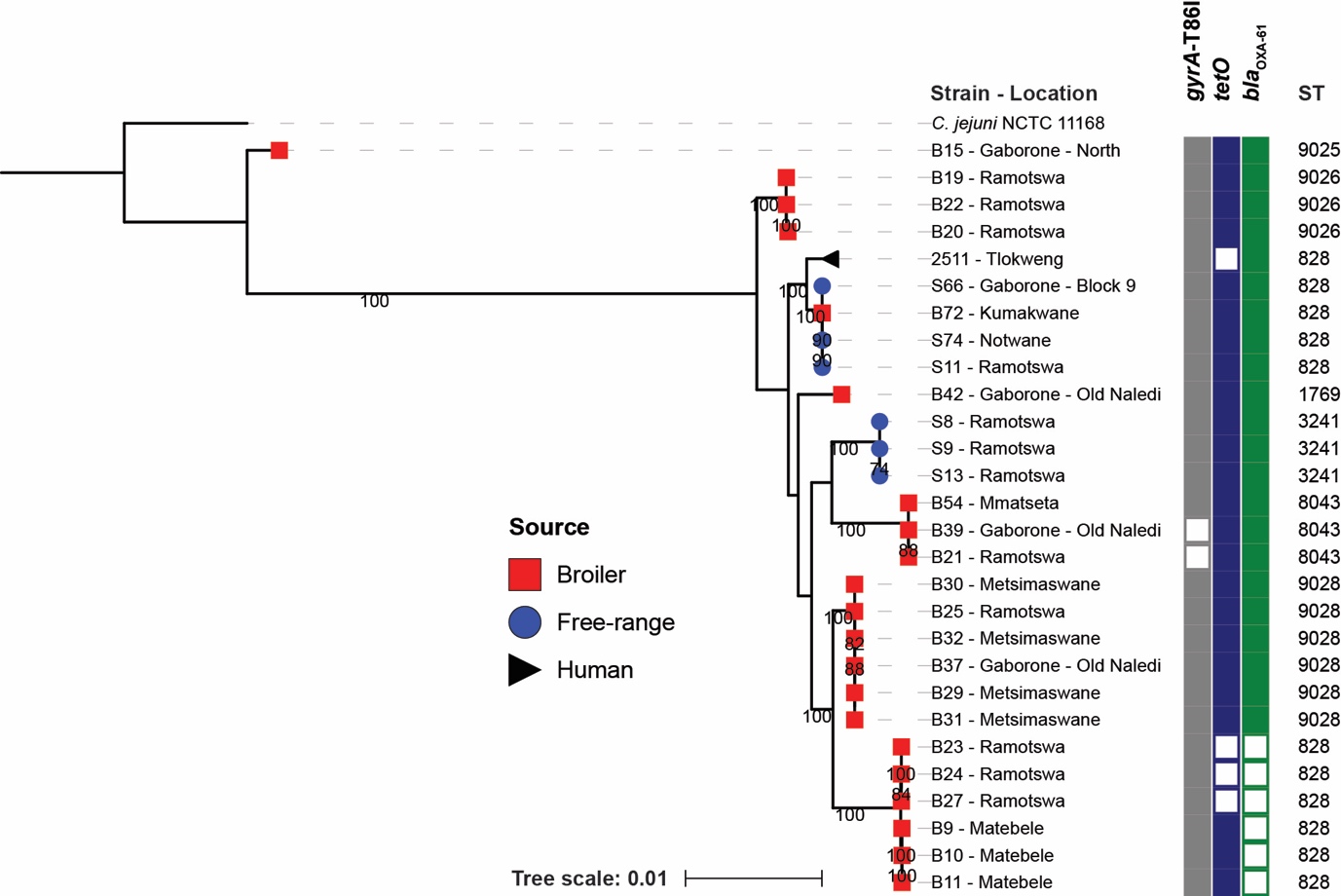
**

S2 Fig. SNP-based phylogeny and AMR profile of C. coli isolates. SNP-based maximum likelihood phylogeny of C. coli isolates visualised in interactive Tree of life tool (iTol) [S1]. The tree was rooted on reference isolate C. jejuni NCTC11168 [S2]. Clustering of isolates was found to be in accordance between core genome and SNP-based phylogenies (Fig 2). Clustering of isolates belonging to the same ST was consistent. Shown for each isolate are: isolate identifier, the geographic location of isolation, presence of AMR determinants and ST.

[S1] Letunic I, Bork P. 2016. Interactive tree of life (iTOL) v3: an online tool for the display and annotation of phylogenetic and other trees. Nucleic Acids Res 44:W242-245.

[S2] Gundogdu O, Bentley SD, Holden MT, Parkhill J, Dorrell N, Wren BW. 2007. Re-annotation and re-analysis of the Campylobacter jejuni NCTC11168 genome sequence. BMC Genomics 8:162.
